# First mitochondrial genomes of Capitellidae and Opheliidae (Annelida) and their phylogenetic placement

**DOI:** 10.1080/23802359.2022.2056537

**Published:** 2022-04-01

**Authors:** Genki Kobayashi, Hajime Itoh, Nobuyoshi Nakajima

**Affiliations:** a Seto Marine Biological Laboratory, Field Science Education and Research Center, Kyoto University, Nishimuro, Japan; b National Institute for Environmental Studies, Tsukuba, Japan

**Keywords:** Capitellida, Opheliida, polychaete, mitogenome, Sedentaria

## Abstract

The complete mitochondrial genomes of *Notomastus* sp. (15,776 bp) (Annelida: Capitellidae) and *Armandia* sp. (18,538 bp) (Annelida: Opheliidae) were assembled for the first time. A group II intron (303 bp) was found in *cox1* of *Notomastus* sp. A phylogenetic analysis revealed that *Notomastus* sp. and *Armandia* sp. were monophyletic, and this clade was clustered with echiurans, although the possibility of the effect of long-branch attraction should be considered.

Annelida is an ecologically diverse group with over 20,000 described species (Capa and Hutchings 2021). Sedentaria includes derivative annelid species that inhabit sediment and possess a cylindrical body with reduced parapodia. Although mitogenomes are often used for inferring the phylogenetic relationships of closely related annelids (Sun et al. 2021), several annelid families are yet undetermined. In this study, we determined the mitogenomes of two families of Sedentaria, namely Capitellidae and Opheliidae, for the first time, examined the features of mitogenomes, and reconstructed their phylogenetic relationships.

The specimens were collected from the intertidal zone in Wakayama, Japan (33°41′N, 135°21′E–135°22′E) and deposited at the Rishiri Town Museum (voucher numbers: RTManl68 (*Notomastus* sp.) and RTManl69 (*Armandia* sp.); contact person Shinri Tomioka, rishiritownmuseum@town.rishiri.hokkaido.jp). DNA extraction and long polymerase chain reaction (PCR) procedures were performed according to the methods used by Kobayashi et al. (2021). Primer sets were designed for each species to amplify nearly complete mitogenomes of *Notomastus* sp. and *Armandia* sp. based on their partial 16S sequences, which were determined using 16SarL/16SbrH (Palumbi 1996) (*Notomastus* sp.) or 16Sann-f2/16Sann-r2 (Kobayashi and Kojima 2021) (*Armandia* sp.). Paired-end sequencing (2 × 300 bp) of the mitogenome amplicons was performed using an Illumina Miseq System (Illumina) at the National Institute for Environmental Studies, Japan. Mitogenomes were assembled with GetOrganelle v1.7.1a (Jin et al. 2020) using the 16S sequences of each species as seed sequences. Assembled contigs and the 16S gene sequence were manually concatenated and the complete mitogenome of *Notomastus* sp. was obtained. The second assembly was conducted for *Armandia* sp. using the *nad5* gene sequence, which was detected with the MITOS2 web server (Donath et al. 2019) from the contig of the first assembly, as a seed sequence, since only a partial mitogenome was obtained from the first assembly. The partial 16S sequence of *Armandia* sp., which was not determined by the above method, was confirmed by Sanger sequencing using 16Sar/16Sb-Arm (16Sb-Arm: 5'-CGYCGGTCTRAACTCAGCTC-3'; this study). Then, the complete mitogenome of *Armandia* sp. was obtained by concatenating the 16S sequence and the contigs obtained in the second assembly. The PCGs and tRNAs were identified using the MITOS2 web server. The tRNAs were also detected using ARWEN (Laslett and Canback 2008) implemented in ARAGORN (Laslett and Canback 2004). The clover-leaf secondary structures of tRNAs were validated using ARWEN. The annotated mitogenome sequences were deposited in GenBank through DNA Data Bank of Japan with accession numbers LC661358 (*Notomastus* sp.) and LC661359 (*Armandia* sp.).

Maximum likelihood phylogeny was reconstructed using amino acid sequences of 13 PCGs according to Kobayashi et al.’s (2021) procedure using the following softwares: IQ-TREE v1.6.12 (Nguyen et al. 2015) for phylogenetic analysis, ModelFinder (Kalyaanamoorthy et al. 2017) for selecting the best-fit substitution models for each of 13 PCGs, SeqKit (Shen et al. 2016) for translating nucleotide sequences, MAFFT v7 (Katoh and Standley 2013) for sequence alignment, and FigTree v1.4.3 (http://tree.bio.ed.ac.uk/software/figtree/) for illustrating the phylogenetic tree. A dataset consisting of 41 mitogenome sequences of Sedentaria and 2 outgroups (Siboglinidae) was obtained from GenBank.

The complete mitogenomes of *Notomastus* sp. and *Armandia* sp. consisted of 15,776 bp (AT content = 57.8%) and 18,538 bp (AT = 60.2%), respectively. Thirteen PCGs and two rRNAs were annotated for both species. *Notomastus* sp. possesses 24 tRNAs and *Armandia* sp. possesses 23 tRNAs, including a tRNA corresponding to the stop codon (anticodon: TTA). Both the species have tRNAs coded on the negative strand (*trnS2* and *trnD* in *Notomastus* sp. and *trnStop* in *Armandia* sp.), which is unusual in Annelida (see Daffe et al. 2021). An intron (303 bp) was found in *cox1* of *Notomastus* sp. based on the alignment of the dataset. This intron was regarded as a group II intron since it included a sequence (starting with GTGCG and ending with AG) similar to the motif of group II intron (starting with GUGYG and ending with AY) (Bonen and Vogel 2001). This intron was inserted in a unique position (1115–1417) and was shorter than other known annelid group II introns in the *cox1* region (Vallès et al. 2008; Richter et al. 2015; Bernardino et al. 2017; Kobayashi et al. 2022). The gene orders of PCGs (Figure 1) were unique for each species among annelids (see Sun et al. 2021). The resultant tree revealed that *Notomastus* sp. and *Armandia* sp. were monophyletic, and this clade was clustered with the echiuran species of *Urechis* (Thalassematidae) (Figure 1). This result was inconsistent with that of previous studies that have shown a sister relationship between echiurans and capitellids (e.g. Struck et al. 2007). Although the close relationship between *Notomastus* sp. and *Armandia* sp. in this study might be affected by long-branch attraction, TreeShrink v1.3.9 (Mai and Mirarab 2018) did not identify any abnormally long branches (the option − q was set to ‘0.05 or 0.10’).

**Figure 1.**
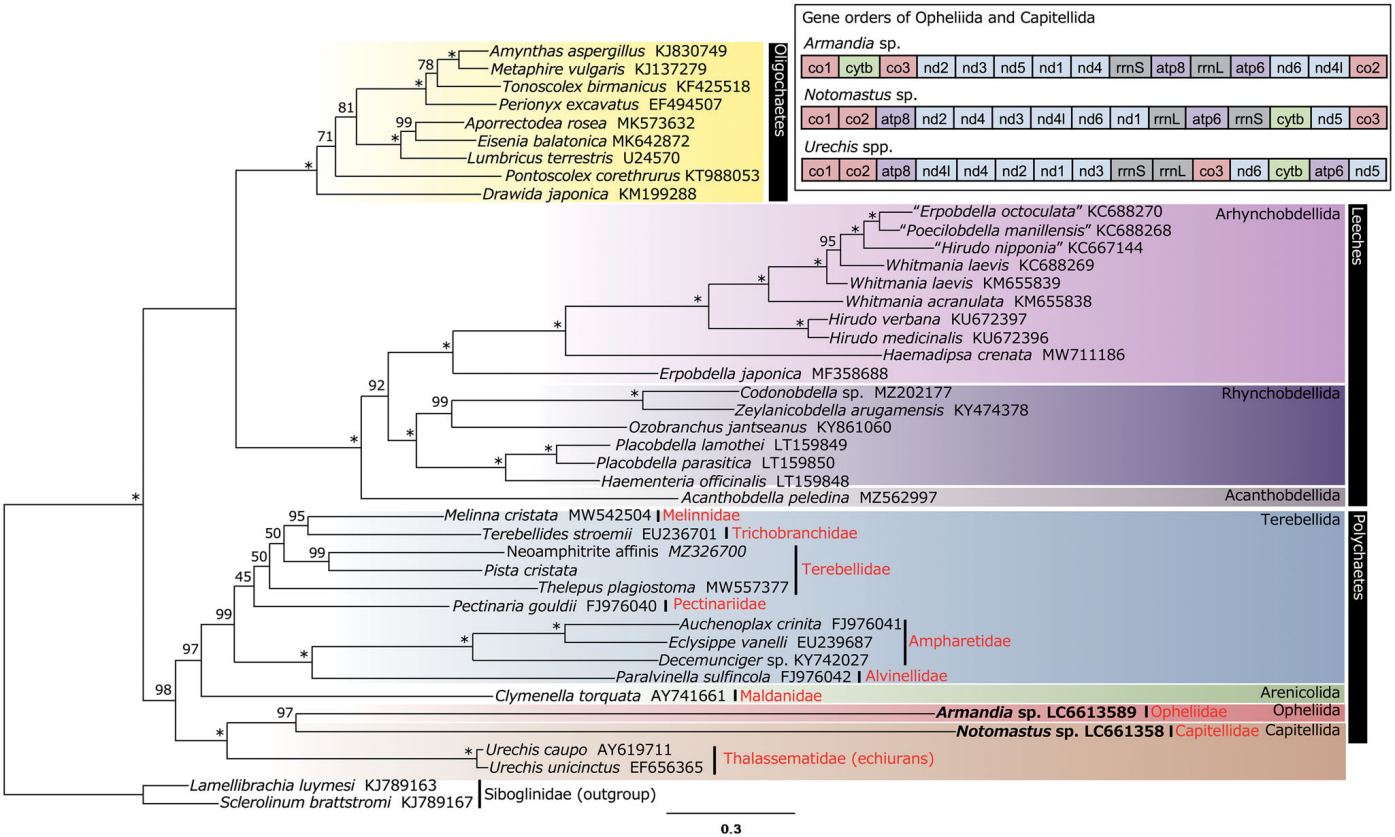
Maximum likelihood phylogeny of a subset of Sedentaria based on the amino acid sequences of 13 mitochondrial genome PCGs (4185 characters). The best-fit substitution models for each of the PCGs were selected by ModelFinder using the Bayesian Information Criterion as follows: mtART + I+G4 (*nad1*), mtInv + F+G4 (*atp6*), mtInv + F + I + G4 (*nad2*, *nad4*, and *nad5*), mtMet + G4 (*nad6*), mtMet + I+G4 (*nad3* and *nad4l*), mtZOA + R4 (*cox1*); mtZOA + R5 (*cytb*), mtZOA + I+G4 (*cox2*), and mtZOA + G4 (*cox3*). Asterisks indicate Bootstrap value = 100%. Gene orders of the PCGs and rRNAs of Capitellida and Opheliida are shown above the tree. The orders of polychaetes and the family of echiurans follow the classification of Struck (2019) and Goto et al. (2020), respectively.

## Authors’ contributions

GK conceived the study. All authors were involved in the analysis and interpretation of the data; the drafting of the paper, revising it critically for intellectual content; and the final approval of the version to be published; and that all authors agree to be accountable for all aspects of the work.

## Data Availability

The sequencing data that support the findings of this study are openly available in GenBank of NCBI under the accession no. LC661358 (*Notomastus* sp.) and LC661359 (*Armandia* sp.). The associated BioProject, SRA, and BioSample numbers are PRJDB12658, DRA013119, and SAMD00428326 (*Notomastus* sp.) and PRJDB12659, DRA013120, SAMD00428327 (*Armandia* sp.), respectively.
